# Non-invasive monitoring of mitochondrial oxygenation and respiration in critical illness using a novel technique

**DOI:** 10.1186/s13054-015-1056-9

**Published:** 2015-09-22

**Authors:** Floor A. Harms, Sander I. A. Bodmer, Nicolaas J.H. Raat, Egbert G. Mik

**Affiliations:** Department of Anesthesiology, Laboratory of Experimental Anesthesiology, Erasmus University Medical Center Rotterdam, ‘s-Gravendijkwal 230, 3015 CE Rotterdam, The Netherlands; Department of Intensive Care, Erasmus University Medical Center Rotterdam, ‘s-Gravendijkwal 230, 3015 CE Rotterdam, The Netherlands

## Abstract

**Introduction:**

Although mitochondrial dysfunction is proposed to be involved in the pathophysiology of sepsis, conflicting results are reported. Variation in methods used to assess mitochondrial function might contribute to this controversy. A non-invasive method for monitoring mitochondrial function might help overcome this limitation. Therefore, this study explores the possibility of *in vivo* monitoring of mitochondrial oxygen tension (mitoPO_2_) and local mitochondrial oxygen consumptionin in an endotoxin-induced septic animal model.

**Methods:**

Animals (rats *n* = 28) were assigned to a control group (no treatment), or to receive lipopolysaccharide without fluid resuscitation (LPS-NR) or lipopolysaccharide plus fluid resuscitation (LPS-FR). Sepsis was induced by intravenous LPS injection (1.6 mg/kg during 10 min), fluid resuscitation was performed by continuous infusion of a colloid solution, 7 ml kg^−1^ h^−1^ and a 2-ml bolus of the same colloid solution. MitoPO_2_ and ODR were measured by means of the protoporphyrin IX-triplet state lifetime technique (PpIX-TSLT). Kinetic aspects of the drop in mitoPO_2_ were recorded during 60s of skin compression. ODR was derived from the slope of the mitoPO_2_ oxygen disappearance curve. Measurements were made before and 3 h after induction of sepsis.

**Results:**

At baseline (t0) all rats were hemodynamically stable. After LPS induction (t1), significant (*p* < 0.05) hemodynamic changes were observed in both LPS groups. At t0, mitoPO_2_ and ODR were 59 ± 1 mmHg, 64 ± 3 mmHg, 68 ± 4 mmHg and 5.0 ± 0.3 mmHg s^−1^, 5.3 ± 0.5 mmHg s^−1^, 5.7 ± 0.5 mmHg s^−1^ in the control, LPS-FR and LPS-NR groups, respectively; at t1 these values were 58 ± 5 mmHg, 50 ± 2.3 mmHg, 30 ± 3.3 mmHg and 4.5 ± 0.5 mmHg s^−1^, 3.3 ± 0.3 mmHg s^−1^, 1.8 ± 0.3 mmHg s^−1^, respectively. At t1, only mitoPO_2_ showed a significant difference between the controls and LPS-NR. In contrast, at t1 both LPS groups showed a significantly lower ODR compared to controls.

**Conclusion:**

These data show the feasibility to monitor alterations in mitochondrial oxygen consumption *in vivo* by PpIX-TSLT in a septic rat model. These results may contribute to the development of a clinical device to monitor mitochondrial function in the critically ill.

## Introduction

Sepsis and septic shock are life-threatening disorders with a high mortality rate of 30–60 % [1]. The pathophysiology of sepsis is multifactorial and complex. Consequently, the treatment of severe sepsis and septic shock requires extensive knowledge and often reaches the limits of possibilities in the ICU. Part of the current treatment for sepsis is aimed at safeguarding or restoring oxygen supply to tissue cells by early aggressive administration of intravenous fluids, and the use of inotropic and vasoactive agents. Early aggressive resuscitation in sepsis is known to modulate inflammation [2] and improve microvascular perfusion [3], even though the clinical outcome is not always improved [4–6]. The management of sepsis therefore remains a challenge despite advances in both monitoring and treatment.

Mitochondrial dysfunction is suggested to be a key issue in the pathophysiology of and recovery from sepsis [7, 8]. In the case of mitochondrial dysfunction, optimization of the macrocirculation and microcirculation alone is not likely to result in improved aerobic cell metabolism. This might explain why treatment focused on adequate tissue perfusion and oxygenation does not always lead to a better prognosis. Despite many studies on the role of mitochondrial dysfunction in sepsis over the last 40 years, clear evidence for the underlying mechanism is still lacking [9]. Whereas some authors report decreased mitochondrial oxygen consumption in sepsis [9–13], others found an unchanged [14] or even improved [15, 16] mitochondrial function under similar circumstances.

Most knowledge on mitochondrial dysfunction in sepsis is derived from animal experiments using isolated cells or mitochondria from tissue biopsies [8, 10, 17], providing detailed insight into the function of the respiratory chain. However, measurements in isolated mitochondria may not reflect the in vivo situation. This is due to the possible loss of essential metabolites during mitochondrial isolation and disruption of the normal interactions of the organelle with the cytoskeleton [18], a situation that is partly solved by applying respirometry on homogenates or permeabilized cells. However, such ex vivo measurements pose experimental limitations that may partly explain the controversial reports on mitochondrial dysfunction in sepsis [9]. In addition, the use of different methods to measure mitochondrial oxygen consumption, different models of sepsis, a long or short duration of sepsis, and different target organs may also contribute to the lack of a clear pattern [19]. Therefore, in their review, Jeger et al. [9] concluded that “… in order to get a better understanding about mitochondrial dysfunction in sepsis a valid non-invasive method to monitor mitochondrial function in vivo would be necessary”.

A method developed to measure the mitochondrial oxygen tension (mitoPO_2_) using the protoporphyrin IX-triplet state lifetime technique (PpIX-TSLT) [20, 21] provides new opportunities to monitor mitochondrial function in vivo. The PpIX-TSLT enables mitoPO_2_ measurements by means of the oxygen-dependent optical properties of 5-aminolevulinic acid (ALA)-induced endogenous protoporphyrin IX (PpIX). Our group investigates mitochondrial respirometry with the aim to monitor mitoPO_2_ and local mitochondrial oxygen consumption measured by the oxygen disappearance rate (ODR) in skin [22–24]. The PpIX-TSLT is the first technique to allow measurement of the mitoPO_2_ in living cells, and can also be applied in vivo [21, 25]. Moreover, because this technique is noninvasive and safe for application in humans [26], the PpIX-TSLT may enable clinical monitoring of mitochondrial function at the cellular level.

The present study explores the possibility to monitor in vivo the mitoPO_2_ and ODR in an animal model of acute critical illness. We report on the use of the PpIX-TSLT for cutaneous respirometry in healthy rats compared with rats with endotoxin-induced sepsis.

## Methods

### Principle of mitoPO_2_ measurements

**Fig. 1:**
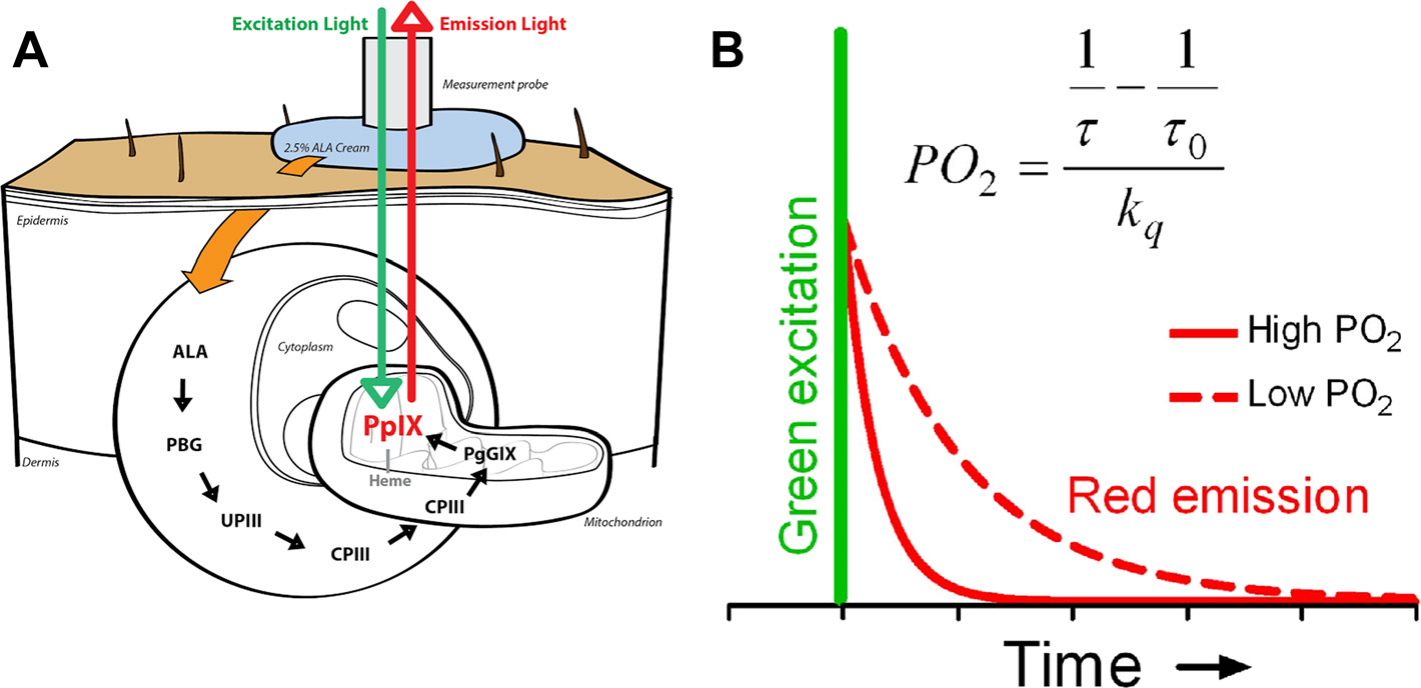
**a** Principle of the PpIX-TSLT. The pathway by which topical ALA administration enhances mitochondrial PpIX levels and the principle of delayed fluorescence detection after an excitation pulse with green (510 nm) light. Emission light is the delayed fluorescence (red light, 630–700 nm) and its lifetime is oxygen dependent. **b** PpIX emits delayed fluorescence after excitation by a pulse of green (510 nm) light. The delayed fluorescence lifetime is oxygen dependent according to the Stern–Volmer equation (inset), in which *k*_*q*_ is the quenching constant and τ_0_ is the lifetime at zero oxygen. *ALA* 5-aminolevulinic acid, *CPIII* coporporphyrinogen III, *PBG* porphobilinogen, *PO*_*2*_ oxygen tension, *PpIX* protoporphyrin IX, *UPIII* urporphyrinogen III

The background of the PpIX-TSLT is described in detail elsewhere [20–22, 24]. In short, PpIX is the final precursor of heme in the heme biosynthetic pathway. PpIX is synthesized in the mitochondria, and administration of ALA substantially enhances the PpIX concentration. Since the conversion of PpIX to heme is a rate-limiting step, administration of ALA causes accumulation of PpIX inside the mitochondria (Fig. 1). PpIX possesses a triplet state that reacts strongly with oxygen, making its lifetime oxygen dependent. Population of the first excited triplet state occurs upon photoexcitation with a pulse of light, and causes the emission of red delayed fluorescence. The delayed fluorescence lifetime is related to the mitoPO_2_ according to the Stern–Volmer equation:

1 $$ P{O}_2=\frac{\frac{1}{\tau }-\frac{1}{\tau_0}}{k_q} $$

in which τ is the measured delayed fluorescence lifetime, *k*_*q*_ is the quenching constant, and τ_0_ is the lifetime at zero oxygen. In the case of a nonhomogeneous oxygen distribution inside the measurement volume, a reliable estimation of the average PO_2_ can be made by the rectangular distribution method (RDM) [27, 28].

### Delayed luminescence setup

A compact computer-controlled tunable laser (Opolette 355-I; Opotek, Carlsbad, CA, USA), providing pulses with a specified duration of 4–10 nanoseconds and typically 2–4 mJ/pulse over the tunable range of 410–670 nm, was used as excitation source. The laser was coupled into a Fiber Delivery System (Opotek) consisting of a 50 mm planoconvex lens, an X–Y fiber mount, and a 2 m fiber with a core diameter of 1000 μm. This fiber was coupled to a custom-made reflection probe by an In-Line Fiber Optic Attenuator (FOA-Inline; Avantes b.v., Eerbeek, the Netherlands). The reflection probe consisted of two 1000 μm fibers with a length of 2 m (P1000-2-VIS-NIR; Ocean Optics, Dunedin, FL, USA) mounted at the common end into a stainless steel holder with a separation of 1 mm between the fibers. The common end of the reflection probe consisted of an aluminum rod with a length of 5 cm and a diameter of 10 mm. The light output of the excitation branch was measured by a FieldMate laser power meter with a PowerMax PS19 measuring head (Coherent Inc., Santa Clara, CA, USA). Our experiments used an excitation energy of 250 μJ/pulse and a repetition frequency of 1 Hz. The PpIX signal was detected by a gated microchannel plate photomultiplier tube (MCP-PMT R5916U series; Hamamatsu Photonics, Hamamatsu, Japan). The emission branch of the reflection probe was fit into an Oriel Fiber Bundle Focusing Assembly (Model 77799; Newport, Irvine, CA, USA) which was coupled to the MCP-PMT by an in-house built optics consisting of a filter-holder, a plano convex lens (BK-7; OptoSigma, Santa Ana, CA, USA) with focal length of 90 mm, and an electronic shutter (04 UTS 203; Melles Griot, Albuquerque, NM, USA). The PpIX emission light was filtered by a combination of a 590 nm longpass filter (OG590; Newport) and a broadband (675–25 nm) bandpass filter (Omega Optical, Brattleboro, VT, USA).

### Principle of the local oxygen consumption measurements

**Fig. 2:**
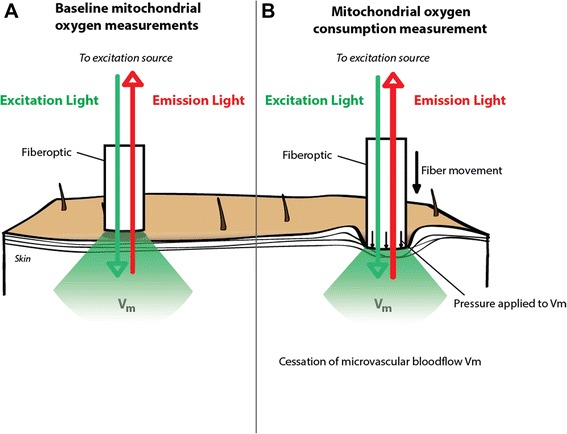
Principles of respirometry in the skin by oxygen-dependent quenching of delayed fluorescence of PpIX. **a** Baseline measurement. **b** Principle of the pressure-induced cessation of microvascular blood flow. *V*_*m*_ measurement volume

The local oxygen consumption is measured by the ODR after local cessation of the oxygen supply by pressure-induced occlusion of the microcirculation. The reflection probe was mounted above the ALA cream-treated skin with a height-adjustable stand, allowing different settings for the probe-to-skin distance. Occlusion of the microcirculation in the skin was obtained by local pressure with the measurement probe. This simple procedure reliably created stop-flow conditions and induced a measurable ODR, due to cessation of the microvascular oxygen supply and ongoing cellular oxygen consumption. The mitoPO_2_ was measured before and during application of pressure at an interval of 1 Hz, using one laser pulse per measurement. The principles of ODR measurements are shown in Fig. 2. We have described these principles in detail and provided a working implementation of the technique for ODR measurements previously [26]. In that implementation, we developed a method for the analysis of delayed-fluorescence-based ODR data to calculate the mitoPO_2_, ODR, and P50 and demonstrated the feasibility and reproducibility of our method in rats [26].

### Animal preparation

The protocol was approved by the Animal Research Committee of the Erasmus University Medical Center Rotterdam, the Netherlands. Animal care and handling were performed in accordance with the guidelines for Institutional and Animal Care and Use Committees.

A total of 28 male Wistar rats (mean body weight 292 g, standard deviation ± 25.5 g) were used in this study. All animals were anesthetized by intraperitoneal injection of a mixture of ketamine 90 mg/kg (Alfasan, Woerden, the Netherlands), 0.5 mg/kg medetomidine (Sedator Eurovet Animal Health BV, Bladel, the Netherlands), and 0.05 mg/kg atropine (Centrofarm Services BV, Etten-Leur, the Netherlands). Ketamine (50 mg/kg/hour) and crystalloid solution (Ringer’s lactate, 5 ml/kg/hour) were infused intravenously to maintain anesthesia and fluid balance. Following tracheotomy, mechanical ventilation was instigated (Babylog 8000 plus; Dräger, Lubeck, Germany). Ventilation was adjusted on end-tidal carbon dioxide tension, keeping the arterial carbon dioxide tension at 35–45 mmHg. The inspired oxygen concentration was set at 40 %. A polyethylene catheter (outer diameter 0.9 mm) was inserted into the right jugular vein for intravenous administration of a colloid solution (Voluven 3.5 ml/kg/hour). Arterial blood pressure and heart rate were monitored with a similar catheter in the left femoral artery. Every hour we performed analysis of arterial blood gas, and measured cardiac output by thermodilution (PowerLab; ADInstruments, Colorado Springs, CO, USA) with the thermistor inserted in the right carotid artery. Body temperature was rectally measured and maintained at 38 ± 0.5 °C by means of a heating pad.

### Experimental setup

In all groups PpIX was induced by topical application of 2.5 % ALA cream. The ALA cream was prepared by mixing hydrophilic cremor lanette (Lanettecreme I FNA; Bipharma, Weesp, the Netherlands) and 2.5 % ALA (Sigma-Aldrich, St. Louis, MO, USA) just before use. The cream was topically administered on the abdominal skin after hair removal. The latter was accomplished by shaving and subsequent use of hair removal cream (Veet; Reckitt Benckiser Co., Slough, UK). The ALA-treated skin was covered with an adhesive film to avoid evaporation. Furthermore, to prevent premature exposure of the primed skin to light, the primed area was covered with aluminum foil. After 3 hours, sufficient PpIX had accumulated to commence baseline (t0) in vivo measurements of the mitoPO_2_ and ODR. All mitoPO_2_ and ODR measurements were performed in a dark environment to prevent ambient light contamination.

After measurements at t0, animals were assigned to one of three groups: lipopolysaccharide without fluid resuscitation (LPS-NR, *n* = 10), LPS with fluid resuscitation (LPS-FR, *n* = 10), and untreated control (*n* = 8). The LPS groups received an intravenous LPS injection (1.6 mg/kg during 10 minutes) (extracted from *Escherichia coli*, serotype 0127:B8; Sigma-Aldrich). The LPS was dissolved at a concentration of 1 mg/ml in a crystalloid solution. The oxygen measurements were repeated 3 h after infusion of LPS (t1).

**Fig. 3:**
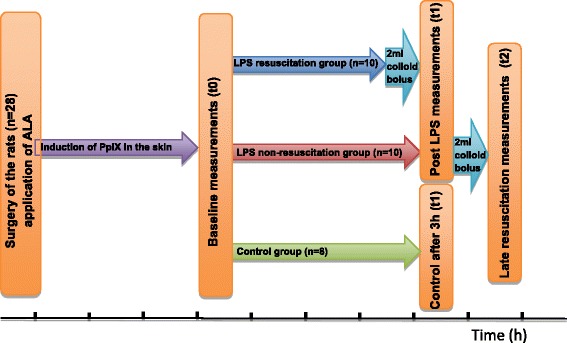
Schematic flow chart of the experimental setup. *ALA* 5-aminolevulinic acid, *LPS* lipopolysaccharide, *PpIX* protoporphyrin IX

In the LPS-FR group, fluid resuscitation was achieved by continuous infusion of a colloid solution at 7 ml/kg/hour (Voluven; Fresenius Kabi, Bad Homburg, Germany); a 2 ml bolus of the same colloid solution was administered just before the start of the t2 measurements. Finally, in the LPS-NR group a 2 ml colloid fluid bolus (Voluven; Fresenius Kabi) was given directly after t1 measurements to achieve a late resuscitation model (LPS-LR). The effect of this late resuscitation on the mitoPO_2_ and ODR was measured directly after fluid administration (t2). Figure 3 describes the experimental setup.

### Statistical analysis

Statistical analyses were performed using IBM SPSS version 21 (IBM Corporation, Armonk, NY, USA). Unless stated otherwise, all values are reported as mean ± standard error.

Statistical significance between groups was calculated using a two-tailed Student's *t* test. A paired Student’s *t* test was used to compare differences between measurements at t0 and t1. *p* ≤0.05 was considered statistically significant.

## Results

Delayed fluorescence signals were easily detected 3 hours after induction with topically applied ALA. Table 1 presents data on the hemodynamic parameters of the experimental groups. At the t0 measurement, all rats were hemodynamically stable without signs of hypoperfusion (reflected by low serum lactate) and there were no significant differences between the groups. At t1, significant (*p* <0.05) changes in hemodynamics were observed in both LPS groups. In the LPS-FR group macrohemodynamic parameters were preserved by means of fluid resuscitation, indicated by unchanged mean arterial blood pressure and cardiac output. In contrast, macrohemodynamic parameters were compromised in the LPS-NR group. Both LPS groups showed significant metabolic acidosis with increased serum lactate.

**Table 1:** Hemodynamic parameters

Parameter	Baseline measurements (t0)	Measurements 3 hours after LPS infusion (t1)	Measurements after late resuscitation (t2)
Control group (*n* = 8)			
Cardiac output (ml/minute)	137 ± 21	115 ± 32	
Mean arterial pressure (mmHg)	78 ± 4	66 ± 3^*^	
Heart rate (bpm)	235 ± 12	244 ± 12	
pH	7.33 ± 0.01	7.28 ± 0.04	
pCO_2_ (mmHg)	39 ± 1.8	36 ± 2.0	
pO_2_ (mmHg)	201 ± 3.99	201 ± 6.64	
	Baseline measurements (t0)	Post-LPS measurements (t1)	
LPS resuscitation group (*n* = 10)			
Cardiac output (ml/minute)	118 ± 28	111 ± 36	
Mean arterial pressure (mmHg)	82 ± 3	80 ± 8	
Heart rate (bpm)	240 ± 7	292 ± 14^*†^	
pH	7.34 ± 0.01	7.16 ± 0.02^*†^	
pCO_2_ (mmHg)	36 ± 2	38.5 ± 2	
pO_2_ (mmHg)	196 ± 5	209 ± 3^*^	
Lactate (mmol/l)	1.10 ± 0.07	3.87 ± 0.32^*†^	
	Baseline measurements (t0)	Post-LPS measurements (t1)	Late resuscitation measurements (t2)
LPS non-resuscitation group (*n* = 10)			
Cardiac output (ml/minute)	135 ± 17	50 ± 19^*^	343 ± 80^*†^
Mean arterial pressure (mmHg)	76 ± 9	57 ± 4	53 ± 4
Heart rate (bpm)	247 ± 10	268 ± 29	294 ± 14
pH	7.35 ± 0.01	7.14 ± 0.01^*†^	7.12 ± 0.01^*†^
pCO_2_ (mmHg)	35 ± 2	37 ± 2	34 ± 3
pO_2_ (mmHg)	192 ± 3	189 ± 7	193 ± 8
Lactate (mmol/l)	1.38 ± 0.10	4.30 ± 0.33^*†^	5.00 ± 0.77^*†^

Data presented as mean ± standard error

^*^Significantly different (*p* <0.05) compared with baseline measurements

^†^Significantly difference (*p* <0.05) compared with control group

*LPS* lipopolysaccharide, *PCO*_*2*_ carbon dioxide tension, *PO*_*2*_ oxygen tension

**Fig. 4:**
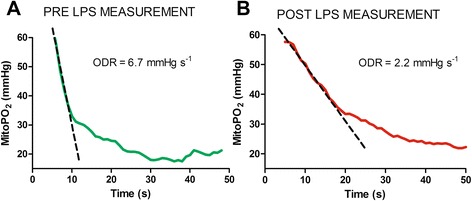
Representative example of mitochondrial oxygen consumption measurement in a rat from the nonresuscitation group. **a** Before administration of LPS. **b** Three hours after administration of LPS. *LPS* lipopolysaccharide, *mitoPO*_*2*_ mitochondrial oxygen tension, *ODR* oxygen disappearance rate

Table 2 presents data on the mitoPO_2_ and ODR. Both parameters showed a significant decrease in the LPS-NR induced septic group, whereas no differences were observed in the control group. In the LPS-FR group, no differences in mitoPO_2_ were observed compared with the control group. However, in spite of normal mitoPO_2_ values, there was a significantly decreased ODR in the LPS-FR group. At t1 in the LPS-NR group, both the mitoPO_2_ and ODR were significantly lower compared with values at t0. Figure 4 presents a representative example of mitochondrial consumption measurements under normal conditions (Fig. 4a) and under septic conditions without resuscitation (Fig. 4b).

**Table 2:** mitoPO_2_ and ODR before and after LPS induction

Parameter	Baseline measurements (t0)	Measurements 3 hours after LPS infusion (t1)
Control group (*n* = 8)		
mitoPO_2_ (mmHg)	59 ± 1	58 ± 5
ODR (mmHg/second)	5.0 ± 0.3	4.5 ± 0.5
	Baseline measurements (t0)	Post-LPS measurements (t1)
LPS resuscitation group (*n* = 10)		
mitoPO_2_ (mmHg)	64 ± 3.0	50 ± 2.3^*^
ODR (mmHg/second)	5.3 ± 0.5	3.3 ± 0.3^*†^
	Baseline measurements (t0)	Post-LPS measurements (t1)
LPS nonresuscitation group (*n* = 10)		
mitoPO_2_ (mmHg)	68 ± 4^†^	30 ± 3.3^*†^
ODR (mmHg/second)	5.7 ± 0.51	1.8 ± 0.3^*†^

Data presented as mean ± standard error

^*^Significant difference (*p* <0.05) compared with baseline measurements

^†^Significant difference (*p* <0.05) compared with control group

*LPS* lipopolysaccharide, *mitoPO*_*2*_ mitochondrial oxygen tension, *ODR* oxygen disappearance rate

**Fig. 5:**
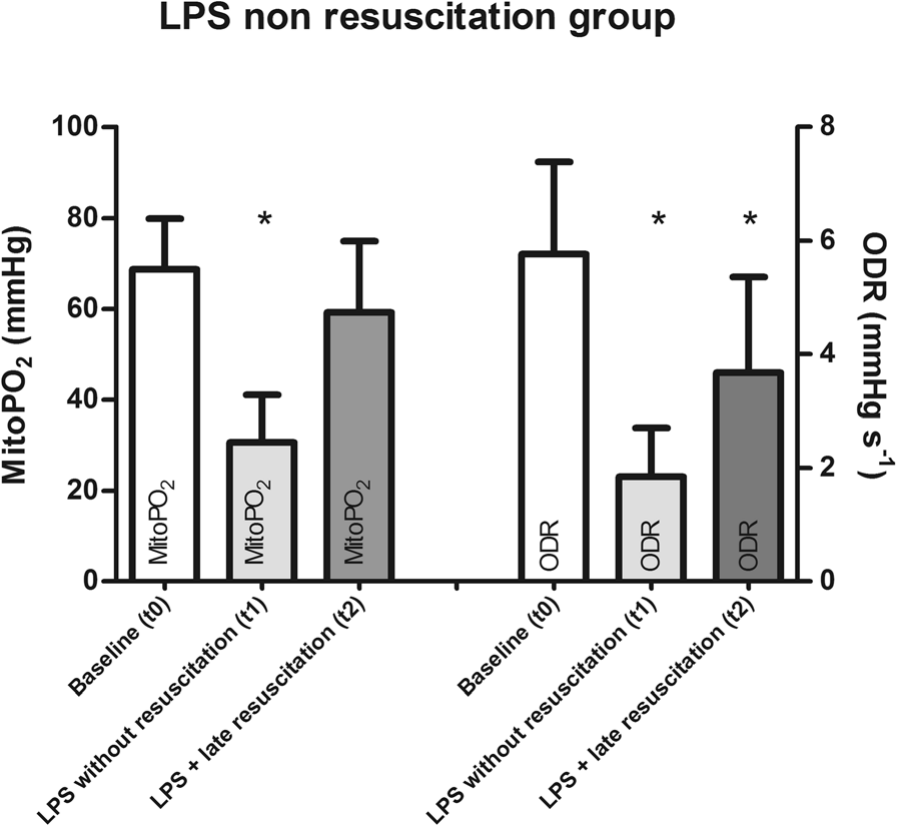
Average mitoPO_2_ and ODR in the nonresuscitation group at three time points: baseline (t0) before the induction of sepsis, 3 hours after LPS infusion (t1), and after late fluid resuscitation (t2). Data presented as mean ± standard error. *Significant difference compared with baseline measurements (*p* <0.05). *LPS* lipopolysaccharide, *mitoPO*_*2*_ mitochondrial oxygen tension, *ODR* mitochondrial oxygen consumption

Figure 5 shows mitoPO_2_ and ODR measurements in the LPS-NR group measured at baseline (t0), during septic conditions without fluid resuscitation (t1), and immediately after a late fluid resuscitation (t2) with a bolus of colloid. The mitoPO_2_ values measured before the stop-flow situation were generally around 65 mmHg; induction of sepsis resulted in hemodynamic changes, with a corresponding decrease in mitoPO_2_ to values around 30 mmHg. The mitoPO_2_ recovered to baseline values after late resuscitation with a fluid bolus. Before late resuscitation, the decreased ODR might be a consequence of the persisting hypoperfusion resulting in low mitoPO_2_ values. Interestingly, however, recovery of the mitoPO_2_ values by fluid resuscitation in the LPS-LR group also failed to result in normalization of ODR.

## Discussion

In this acute model of LPS-induced sepsis in rats, our results support the idea that mitochondrial dysfunction contributes to the pathophysiology of sepsis, even in the absence of severe hemodynamic shock and diminished mitochondrial oxygenation. We found reduced cellular oxygen consumption after restoring tissue oxygenation to baseline levels with fluid resuscitation. Reduced mitochondrial respiration in the absence of hypoxia clearly indicates a hampered aerobic metabolism. Importantly, these measurements were made on the cellular level in vivo, thereby eliminating the potential bias introduced by more invasive and destructive techniques such as mitochondrial isolation.

As expected in an animal model of induced sepsis, our endotoxemic rats were hemodynamically compromised with a low mean arterial pressure and cardiac output. Septic rats also showed high lactate levels and low pH levels, and signs of metabolic acidosis, suggesting a possible increase in anaerobic metabolism. However, while the induction of sepsis without fluid resuscitation resulted in a clear decrease in both the mitoPO_2_ and ODR, fluid resuscitation resulted in other changes. Early fluid resuscitation, given to avoid the development of tissue hypoperfusion, resulted in a decreased ODR while the mitoPO_2_ remained almost unchanged compared with the control group. Furthermore, late resuscitation by a fluid bolus almost completely restored the mitoPO_2_ to baseline values. However, in contrast to the idea that decreased oxygen consumption is secondary to tissue hypoxia, the ODR did not recover after fluid resuscitation.

An impaired ODR, despite a normal mitoPO_2_, suggests persisting mitochondrial dysfunction in the endotoxemic animals. Administration of LPS in rats is known to induce complex I inhibition of the mitochondrial respiratory chain in the heart, liver and diaphragm [14, 29–31]. The present findings are probably based on the same principles and are likely to reflect the in vivo situation of this phenomenon. However, obviously some care should be taken with such a mechanistic interpretation of our results. Also, since the mitoPO_2_ reflects the balance between local oxygen supply and demand [26], a decrease in the ODR could be expected to result in an increase in the mitoPO_2_. Since we did not measure microvascular blood flow we can only speculate on why we did not observe such a phenomenon. Vascular autoregulation normally matches blood flow to metabolism, and under baseline conditions mitoPO_2_ measurements are indeed highly reproducible. In the absence of vascular autoregulation, a high cardiac output in combination with a reduced ODR (the situation after late resuscitation) could indeed be expected to lead to an increased mitoPO_2_ compared with baseline. The fact that the mitoPO_2_ more or less restores to baseline values suggests that in our model the autoregulation in the cutaneous microcirculation is not (yet) severely compromised.

In addition to these findings, our results demonstrate that the PpIX-TSLT enables monitoring of the mitochondrial function in skin. The technique provides in vivo information on oxygenation and oxygen consumption of the mitochondria, without the need to damage tissue for isolation of mitochondria. Therefore, our method overcomes some of the disadvantages of earlier studies on mitochondrial dysfunction in sepsis. Because the PpIX-TSLT can be applied in humans, the technique is potentially feasible for clinical monitoring [32]. Although our implementation of the PpIX-TSLT is an important step towards addressing the need for a valid noninvasive method to monitor mitochondrial function in vivo, as proposed by Jeger et al. [9], some questions still remain. The clinical relevance of our method needs additional studies which, for example, investigate the correlation between mitochondrial function in the skin and other organs.

It is important to note that with the PpIX-TSLT we can measure relatively high mitoPO_2_ values in the skin. In fact, the mitoPO_2_ appears to be in the same range as the intracutaneous oxygen tension measured by micro oxygen electrode measurements [33, 34]. Since oxygen transport into tissue cells is driven by a diffusion gradient, one would expect mitoPO_2_ to be lower, so our data are surprising and somewhat controversial. Unfortunately, a noninvasive gold standard for measuring (intra)cutaneous oxygen tension is lacking. Although the PpIX-TSLT is quantitative and calibrated, the interpretation of the data assumes exclusive mitochondrial localization of the PpIX, which owing to technical limitations has not been conclusively verified to date. Important in this respect is that we did not see delayed fluorescence in full blood and plasma, arguing against intravascular localization of PpIX as a reason for high mitoPO_2_ readings. Furthermore, the PpIX-TSLT shares the property of other lifetime techniques in that it becomes less accurate for shorter lifetimes. The mitoPO_2_ measurements therefore become less robust at higher oxygen tensions. Although our data clearly demonstrate that the PpIX-TSLT is a valuable addition to the arsenal of in vivo oxygen measurements, further evaluation of this novel technique is needed and ongoing. The reader should bear these considerations in mind while interpreting the absolute values of the measurements.

## Conclusions

This study shows the feasibility to measure mitochondrial oxygenation and oxygen consumption in endotoxemic rats by means of a cutaneous PpIX-TSLT. A decreased local oxygen consumption in the presence of preserved or restored mitoPO_2_ suggests that mitochondrial dysfunction contributes to the metabolic failure in sepsis, even in the absence of hemodynamic shock. These results are promising in view of our aim to develop a clinical mitochondrial monitoring technique. Clinical implementation of this technique is likely to contribute to our understanding of mitochondrial dysfunction in sepsis and the development of therapeutic approaches aimed at restoring aerobic metabolism and cellular function.

## Key messages

- The PpIX-TSLT is able to measure mitochondrial oxygenation and oxygen consumption in endotoxemic rats
- The PpIX-TSLT is able to measure changes in mitochondrial oxygenation and oxygen consumption due to the induction of endotoxemia
- The data from this study suggest that mitochondrial dysfunction contributes to the metabolic failure in sepsis

## Abbreviations

ALA: 5-Aminolevulinic acid
LPS: Lipopolysaccharide
LPS-FR: Lipopolysaccharide with fluid resuscitation
LPS-LR: Lipopolysaccharide with late resuscitation
LPS-NR: Lipopolysaccharide without resuscitation
mitoPO_2_: Mitochondrial oxygen tension
ODR: Oxygen disappearance rate
PpIX: Protoporphyrin IX
PpIX-TSLT: Protoporphyrin IX-triplet state lifetime technique
RDM: Rectangular distribution method
